# Crimean-Congo Hemorrhagic Fever, Afghanistan, 2009

**DOI:** 10.3201/eid1710.110061

**Published:** 2011-10

**Authors:** Mir Lais Mustafa, Edris Ayazi, Emad Mohareb, Sam Yingst, Alia Zayed, Cynthia A. Rossi, Randal J. Schoepp, Jawad Mofleh, Kathy Fiekert, Zarif Akhbarian, Homayoon Sadat, Toby Leslie

**Affiliations:** Author affiliations: Afghan Public Health Institute, Kabul, Afghanistan (M.L. Mustafa, E. Ayazi, J. Mofleh, Z. Akhbarian, H. Sadat);; US Naval Medical Research Unit No. 3, Cairo, Egypt (E. Mohareb, A. Zayed);; US Army Medical Research Institute for Infectious Diseases, Fort Detrick, Maryland, USA (S. Yingst, C.A. Rossi, R.J. Schoepp);; Health Protection and Research Organisation, Kabul (K. Fiekert, T. Leslie);; London School of Hygiene and Tropical Medicine, London, UK (T. Leslie)

**Keywords:** viruses, vector-borne infections, ticks, Crimean-Congo hemorrhagic fever, CCHF, Afghanistan, outbreak, seroprevalence, dispatch

## Abstract

In response to an outbreak of Crimean-Congo hemorrhagic fever in western Afghanistan, we measured immunoglobulin G seroprevalence among household members and their animals. Seroprevalence was 11.2% and 75.0% in humans (n = 330) and livestock (n = 132), respectively. Persons with frequent exposure to cattle had an elevated risk of being immunoglobulin G positive.

Crimean-Congo hemorrhagic fever (CCHF) is a vector-borne hemorrhagic disease caused by a primarily zoonotic virus infecting a wide range of domestic and wild animals. The main implicated vectors are *Hyalomma* spp. ticks. Transmission of the virus to humans occurs through tick bites; crushing of infected ticks; contact with blood, body fluids, and tissue of patients with CCHF during the acute phase of illness; and contact with blood or tissue of viremic livestock (*1*). In recent years, several CCHF outbreaks were reported in Afghanistan, and the disease persists in neighboring countries (*2**,**3*). In March 1998, an outbreak with 19 cases and 12 deaths (case fatality rate 63.2%) was reported from Takhar Province in the northern part of the country (*4*).

In the fall of 2008, an outbreak occurred in Herat City, western Afghanistan, with ≈60 suspected cases. CCHF was identified in 6 specimens at the Central Public Health Laboratory in Kabul and later confirmed at the laboratories of the US Naval Medical Research Unit No. 3 (NAMRU-3) in Cairo, Egypt. In August 2009, we conducted a cross-sectional seroprevalence survey among livestock-owning households in the same districts of Herat City where the outbreak occurred.

## The Study

The study was conducted in 100 households of 9 affected villages of Engil District on the edge of Herat City. Households were included if they were located on 3 randomly selected transect lines and if members of the households owned either cattle or sheep. Because there are no reliable estimates on seroprevalence of CCHF in Afghanistan, we estimated prevalence of immunoglobulin (Ig) G to be 7% (*2**,**3*). A sample size of 160 persons was required to detect this prevalence at the 95% confidence level. Allowing for cluster sampling at the household level, 320 persons was the target sample size (≈90 households, assuming an average of 3.5 persons >15 years of age per household). From each household, all members >15 years were surveyed, if they gave consent. Sheep and cattle were selected as livestock types, and 1 or 2 animals per household were randomly selected.

Inclusion criteria for humans were residing in a livestock-owning household, giving informed consent, being >15 years of age, willing to answer the risk factor questionnaire, and willing to give 5 mL of blood. For collection of blood samples and vector specimens from sheep and cattle, permission was given by the head of household.

A standardized, structured, and pretested questionnaire that covered individual data for each participant, including personal details, exposure variables, and self-reported disease history, was used. Animal data were collected from each household and included each animal’s origin, tick exposure, age, and sex. Information was reported by the head of household or owner of the animal(s).

Blood samples from human participants were collected by trained health workers according to standard procedures. A veterinarian collected blood and tick samples from livestock subjects. Blood samples were centrifuged at room temperature at the local laboratory on the day of collection. Serum was separated, frozen, and transported to Kabul for storage and onward shipment. A sandwich/indirect ELISA detected specific IgG at a 1:100 dilution for all human samples by using the VECTOR-BEST diagnostic kit, (VECTOR-BEST, Novosibirsk, Russia). We have previously compared this kit with an in-house ELISA (US Army Medical Research Institute for Infectious Diseases, Fort Detrick, MD, USA), and the results were comparable in all samples tested (L. Mustafa et al., unpub. data). We used an in-house ELISA using the IbAr 10200 strain of CCHF as antigen (US Army Medical Research Institute for Infectious Diseases) and anti-species IgG horseradish peroxidase–conjugated (KPL Inc., Gaithersburg, MD, USA) to test for IgG in all animal serum specimens. All positive samples were confirmed by duplicate testing in a different run. All ELISA testing was performed at NAMRU-3.

Data were analyzed by using Stata version 8 (StataCorp, College Station, TX, USA). The primary outcome was seropositivity among household members, with secondary outcomes being seropositivity in animals and the presence of virus in ticks. Exposure factors for seroprevalence were identified on an a priori basis and appropriate measures of statistical significance were applied to detect differences at the 95% confidence level. The study was approved by the ethics boards of NAMRU-3 and the Afghan Public Health Institute, Afghanistan.

In total, 330 persons were enrolled from 100 households. Among our sample, IgG seroprevalence was 37/330 (11.2%, 95% confidence interval [CI] 8.0–15.1). Of all the potential explanatory variables, only 2 factors were associated with an elevated risk of IgG positivity: daily contact with cattle (33/264 [12.5%] vs. 1/52 [1.8%]; χ^2^ = 5.1, p = 0.02) and exposure to raw animal skins (24/144 [16.7%] vs. 12/176 [6.8%]; χ^2^ = 7.7, p = 0.006). Age group was not associated with seroprevalence.

Self reported clinical illness (fever) occurred in 55% of participants over a 5-month reporting period. Among the participants, 20.8% reported that they had had an illness involving bleeding from teeth, gums, and or other parts of the body, but this event was not associated with IgG positivity or with age group.

These results suggest that the risk for CCHF exposure is uniformly high among the population. The oldest age group shows an approximate lifetime risk for exposure and seroconversion of 17% (95% CI 10.2%–25.8%).

Ninety-two cattle and 40 sheep were included, and serologic analysis of their blood samples was conducted. Seroprevalence was 79.1% (95% CI 69.0%–87.1%) among cattle and 75.0% (95% CI 57.0%–88.5%) among sheep. Prevalence was uniformly high regardless of age, sex, or origin of the animals, suggesting that the disease is highly endemic in the livestock population. Among our sample, 84.6% of cattle and 71.5% of sheep had ticks upon inspection by the surveyors. Ticks (n = 259) from domestic animals were predominantly adult *Hyalomma marginatum* (94.6%). Of the total *Hyalomma* ticks collected, 83% were found on cows. Engorged females were found more on cattle than on sheep (43% and 27%, respectively). No association was found between tick infestation and animal serologic results. Most (>85%) animal owners reportedly control ticks by using pesticides. We did not identify virus in tick specimens by PCR (*5*).

## Conclusions

Seroprevalence in this population of animal owners is higher than in other reported studies from the region (*3**,**4*), and the risk for exposure appears approximately uniform. This finding indicates that universal control measures are required. The route of transmission to humans is either through the bite of ticks or through contact with infected animals or animal products. This second route of transmission is probably more important in Iran and Afghanistan than tick-borne transmission.

Control of CCHF requires control of the disease vector, and surveillance is necessary to ensure optimum timing of interventions such as livestock dipping or sponging because tick abundance is highly seasonal. Further research on rates of antibody acquisition among humans and animals, virus transmission dynamics, and effectiveness of disease control measures is required. CCHF is a regional public health concern of larger than previously acknowledged significance and requires control mechanisms from both the health and agriculture sectors.

## References

[1] Ergönül O. Crimean-Congo haemorrhagic fever. Lancet Infect Dis. 2006;6:203–14. 10.1016/S1473-3099(06)70435-2 16554245 PMC7185836

[2] Athar MN, Bagai HZ, Ahmad M, Khalid MA, Bashir N, Ahmad AM, Crimean-Congo hemorrhagic fever outbreak in Rawalpindi, Pakistan, February 2002. Am J Trop Med Hyg. 2003;69:284–7. 14628945

[3] Izadi S, Holakouie-Naieni K, Majdzadeh SR, Chinikar S, Nadim A, Rakhshani F, Seroprevalence of Crimean-Congo hemorrhagic fever in Sistan-va-Baluchestan province of Iran. Jpn J Infect Dis. 2006;59:326–8. 17060701

[4] World Health Organization. 1998–Crimean-Congo haemorrhagic fever in Afghanistan—outbreak reported. Outbreak News 1998 [cited 2010 Oct 14]. http://www.who.int/csr/don/1998_05_08a/en/index.html

[5] Drosten C, Göttig S, Schilling S, Asper M, Panning M, Schmitz H, 2002, Rapid detection and quantification of RNA of Ebola and Marburg viruses, Lassa virus, Crimean-Congo hemorrhagic fever virus, Rift Valley fever virus, dengue virus and yellow fever virus by real-time reverse transcription-PCR. J Clin Microbiol. 2002;40:2323–30. 10.1128/JCM.40.7.2323-2330.2002 12089242 PMC120575

